# Quality Management in Polish Biobanking Network—Current Status Before the Implementation of Unified and Harmonized Integrated Quality Management System

**DOI:** 10.3389/fmed.2021.780294

**Published:** 2022-01-10

**Authors:** Agnieszka Matera-Witkiewicz, Magdalena Krupińska, Patrycja Sitek, Michał Laskowski, Karolina Zagórska, Joanna Gleńska-Olender

**Affiliations:** ^1^Screening Biological Activity Assays and Collection of Biological Material Laboratory, Faculty of Pharmacy, Wroclaw Medical University Biobank, Wroclaw Medical University, Wroclaw, Poland; ^2^BBMRI.pl Consortium, Wroclaw, Poland

**Keywords:** quality management system-QMS, audits, biobanking, BBMRI.pl, BBMRI-ERIC, harmonization

## Abstract

In 2017, Polish Biobanking Network was established in Poland, within BBMRI.pl project titled “Organization of Polish Biobanking Network within the Biobanking and Biomolecular Resources Research Infrastructure BBMRI-ERIC” as a strategic scientific infrastructure concept. One of the key elements of the project was the verification of the current status of QMS in the Polish biobanking institutions and the implementation of common solutions. The main goal was to indicate the current QMS level and determine the starting points for QMS development for each biobank of the Polish Biobanking Network (PBN). Within 3 years, 35 audit visits were performed. The current status and the level of QMS implementation in each biobank were assessed. Five hundred and seventy recommendations were prepared. The data was analyzed using Fischer Exact test to determine whether or not a significant association was observed. Three areas of analysis were covered: (1) BBMRI.pl status, (2) QMS implementation level and (3) private/public party, respectively. The results were discussed within 15 areas. Concluding remarks showed that some differences were observed in the case of subgroups analysis. There is convergence in QMS within the biobanks where Tissue Banks are located. Moreover, some discrepancies between the QMS implementation level in BBMRI.pl Consortium biobanks and PBN biobanks are observed. Nevertheless, the consortium members are obliged to prepare other biobanks willing to enter the PBN as Members/Observers or which already are in the PBN, so that they can meet the requirements of the quality management system that will enable efficient management of biobanking processes in these units. That is why some actions within BBMRI.pl projects are organized to help the whole biobanking community in Poland implement the harmonized solution.

## Introduction

One of the main goals of biobanking is an increase in the efficiency and excellence of biomedical research, leading to the creation and development of new medical treatments (1). It can be done by facilitating access to quality-well-defined resources such as biological material (BM) samples with associated data. The pre-analytical handling procedures during collection, transport, qualification, processing and storage of BM may directly implicate the occurrence of the most common errors at the analytical level (2–4). Furthermore, it must be pointed out that even the best analytical tools cannot perform reliable, repeatable and suitable results when the samples and associated data quality are insufficient. Pre-analytical processes consist of a series of complex steps that must be performed and supervised using appropriate tools. That guarantees constant monitoring of the obtained effects and ensures their high quality (5). It also finds application in translational medicine, where the results of preclinical studies directly translate into patient therapy. These studies have to be of the highest quality and credibility as they determine the well-being of patients. The key to ensuring the quality of research is working on the highest quality of biological material and data—this is what biobanks are responsible for.

Quality aspects present an increasing trend in biobanking and biomedical issues. Each year, more quality-derived events are observed. Moreover, professional biobanking infrastructures such as BBMRI-ERIC, societies and organizations, including ISBER (6), ESBB and IARC (7), highly promote and constantly develop the areas of quality management within the biobanking community (8, 9).

The effectiveness and credibility of biobanks require the adoption and implementation of optimal standards of practice, including general principles and standard operating procedures (SOP) (10). Standardization is a key factor that regulates and harmonizes the processes within an organization. BBMRI-ERIC, as an observer liaison for the International Organization for Standardization (ISO), contributes to the biobank relevant international standard developments (ISO/TC276 biotechnology and ISO/TC212 clinical laboratory testing and *in vitro* diagnostic test systems) and in bidirectional information exchange by communicating expert knowledge of the ISO working group to the BBMRI-ERIC community. In 2018, the first dedicated standard for Biobanks was published as ISO 20387:2018: *Biotechnology-Biobanking-General requirements for biobanking* (11).

Also within BBMRI.pl project (12), *Quality Standards for Polish Biobanks* (QSPB) were established as common standards for Polish Biobanking Network (PBN) entities (13). Moreover, a unified QMS audit process was invented and consistently implemented.

The main purpose of the work was to check the current state of QMS and the processes taking place in biobanks that belong to PBN. Based on the previous work, where the general audit areas were established, 15 dedicated areas were defined and analyzed (14). As an outcome, the possibility of a detailed analysis of the QMS level implementation in the PBN biobanks was created. The results were compared within three statistical subgroups with regard to BBMRI.pl status (BBMRI.pl consortium member, PBN Member/Observer), the level of QMS implementation and the type of biobank (private/public sector). The results of the work determine further directions for quality management aspects development within BBMRI.pl. Moreover, it will be a significant input for the biobanking community from all organizations interested in the development of biobanking quality aspects, where the Polish Biobanking Network would be an excellent case study example. The systematic audit process within PBN, where the objective assessment was performed, becomes an effective and reliable tool to support biobanking activity and improvement.

## Materials and Methods

### Audit Process

Audits have been carried out in accordance with the requirements of ISO 19011:2018 regarding PERC (*Planning, Execution, Reporting, Close out/down findings*). The objective evidence was collected using observations, documented information and interviews. The assumption of the audit was based on an accessible process called “*friendly audit*”; a similar formula is also presented in BBMRI.de audit system (15). Also the QM audit process is being noticed in other partner countries from BBMRI-ERIC such as Austria (16) and Finland. Audits were performed as on-site and remote meetings. In total, 35 audits were carried out in 2018–2020.

The main scope of the preliminary audit was to encourage biobanks to start cooperation within BBMRI.pl/PBN and to indicate the starting points where the QMS implementation can be focused and started. The auditors have based the audit process on general ISO standards (9000 series: 9001:2015, 9004:2018) where quality aspects for all organizations are collected. The audit begins with an opening meeting conducted by the lead auditor to introduce the audit team and discuss the audit objectives, scope, and program. During the audit, the auditors took audit samples. During the first visit, the auditors got acquainted with the processes taking place in Biobank, documentation of the type of procedures and instructions (if established and implemented in Biobank), records of processes (paper and/or electronic). During the first visit, the auditors familiarize themselves with: the basis of Biobank's operations (e.g., organizational structure of the unit, approval of the Bioethics Committee or other); with protocols/instructions for handling biological material stored in the biorepository (if established); with the way of marking the material (tube/container description, coding, etc.); with the method of recording Biobank resources (paper documentation and/or an Excel list and/or specialized computer software, etc.); with the method of assessing the quality of the tasks performed (if there are intra-laboratory controls or the center takes part in external laboratory controls); with auxiliary processes concerning, inter alia, the process of hiring and training employees, assigning them authorizations, cooperation with suppliers. After the audit, at the closing meeting, the lead auditor presented and discussed the results of the audit, and the recommendations issued. A summary of the Audit report was a list of observations and recommendations for individual areas of QMS covered by the audit. It was planned that the 1st audit report will not contain non-conformities. The implementation and assessment of the effectiveness of the actions taken should be subject to verification, e.g., during the management review. Verification of the reference to the recommendations is also a part of the next audit (audit input no. 2). The background and audit preparation was presented also in our previous paper (14).

### Audited Areas

Cooperation in QM BBMRI-ERIC WGs, the Polish Committee for Standardization in TC 287 Biotechnology and ISO TC 276 WG2 and WG4 provides the best knowledge on standards dedicated to biobanking. The previously determined 13 areas (14) were modified as follows: (1) *Training* was added to *Human Resources Management*, (2) *Traceability of technical and technological processes, Controlled storage process* were combined within one area of *Traceability*, (3) *Strategic and operational objectives* were added to *Quality Management*, (4) *Quality control of deliveries* were changed to *Supplies, material management*, (5) *Monitoring of environmental conditions* and *Handling of hazardous* waste were combined in one area of *Environmental and staff hygiene*. Some other areas were also changed or added, which resulted in the establishment of 15 areas (similar to QSPB) (1) *Management of Biobanks*, (2) *Quality management*, (3) *Documentation and records*, (4) *Human Resources Management*, (5) *Ethical and Legal Aspects-ELSI*, (6) *Supplies, materials management*, (7) *Equipment*, (8) *Traceability*, (9) *Environmental and staff hygiene*, (10) *Biobanking processes and quality control*, (11) *Deviations, non-conforming product/data or service*, (12) *Audits*, (13) *Improvement*, (14) *Biobank cooperation in the scientific, research and development area* and (15) *Safety&Security*.

No non-conformities are revealed in the first audit report. Furthermore, the scope of needs and expectations of each biobank toward BBMRI.pl QMS team was estimated.

### Data Analysis

The obtained data were subject to statistical and descriptive analysis, with division into three main categories: group 1—comparison of Members/Observers from PBN (35 units) and Consortium members (6 units); group 2—the division into public (36 units) and private biobanks (5 units). Public biobanks were operating at universities, research institutes and public hospitals. Private biobanks operated as part of private laboratories; group 3—biobanks with QMS implemented (11 units), biobanks without QMS (30 units), biobanks operating within tissue and cell banks- the entities specialized with the collection, processing, storage and production of human tissues and cells for therapeutic issues (6 units). Units with established and applied integrated QMS were considered as biobanks with QMS implemented, regardless of whether they are ISO 9001 certified or not. Within the groups, specific subgroups were analyzed.

Categorical data were described using the scores for meeting the requirements of the audit area and percentage using the same criteria as described in the previous paper (14). The associations between the fulfillment of the requirements of the audit area in different groups were tested using Fisher's exact test, where non-random associations between two categorical variables are checked. The statistical significance used in the analysis of contingency tables, also known as cross tabulation, was used. The significance level was set at 0.05. The significance values were as follows: ^****^/^***^ extremely significant (*p*-value 0.001–0.001), ^**^ very significant (0.001–0.01), ^*^ significant (0.01–0.05), ns- not significant (>0.05). All statistical analyses and graphs were performed using GraphPad Prism 8.0.1. The results were presented in all figures.

## Results

**Figure 1:**
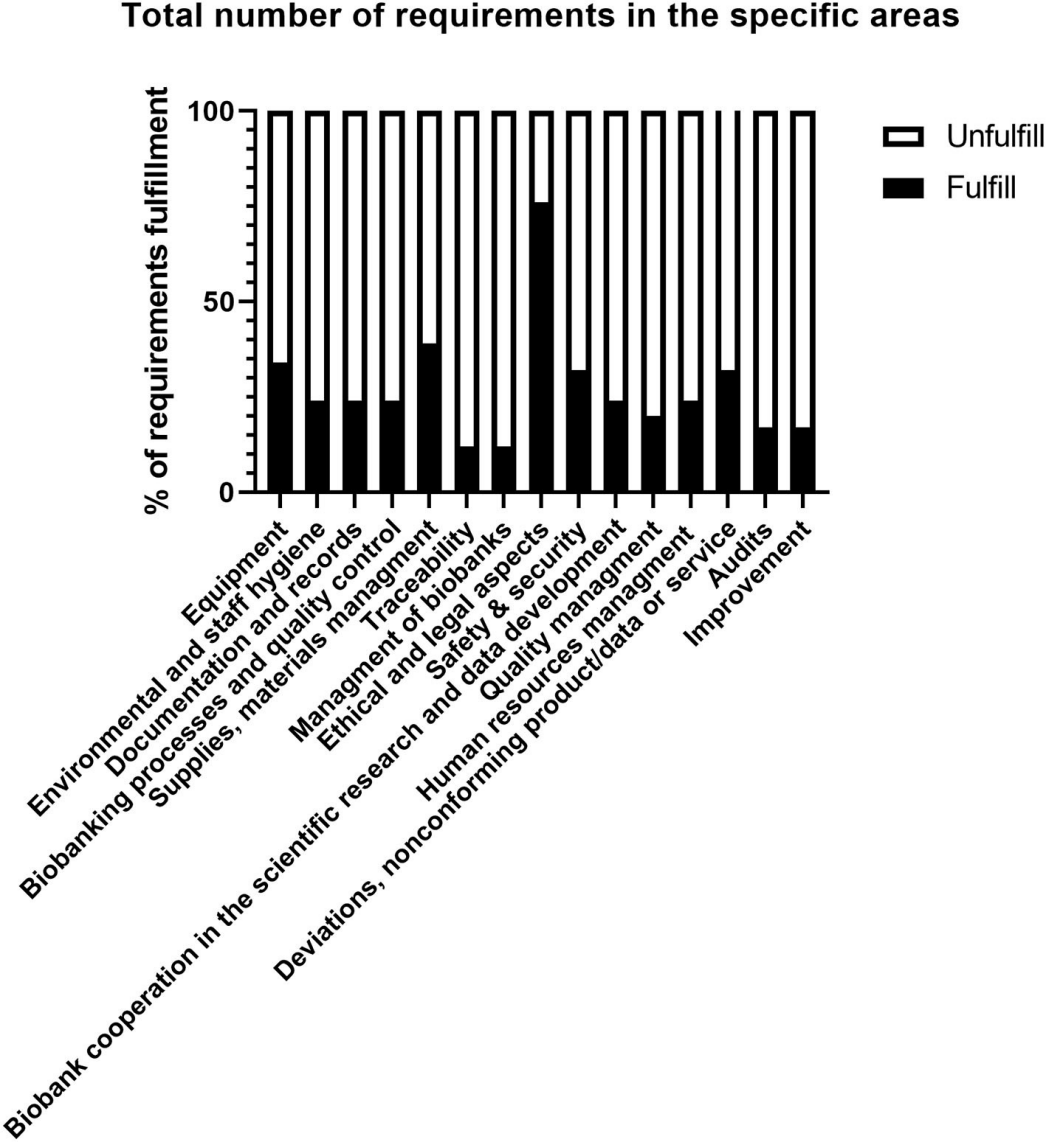
Total number of requirements in the specific areas.

In 2018–2020, 41 audits were performed. The current status and the level of QMS implementation in each biobank were assessed. Figure 1 presents the results for all PBN biobanks. Within the *ELSI*, 76% of biobanks met the requirements. The majority of biobanks possess well-prepared donor's documentation regarding national and international projects. Nevertheless, the most frequently identified deficiencies within the *ELSI* included incorrect IC forms in terms of the donor's rights and freedoms (20%); lack of records regarding the processing of personal data in accordance with the GDPR (16.7%); lack of procedures for obtaining IC (10%). The results show that effective activity is needed in *Traceability* and *Management of Biobanks* (12% fulfillment). Less than half of the biobanks (24%) had implemented the QMS system as well as the procedures and guidelines for biobanking processes, however, sufficient knowledge was presented. Recommendations indicate the lack of ability to quickly identify a critical device involved in the technological process (33.3%) and the lack of records for consumables and reagents used in the process (16.6%).

**Figure 2:**
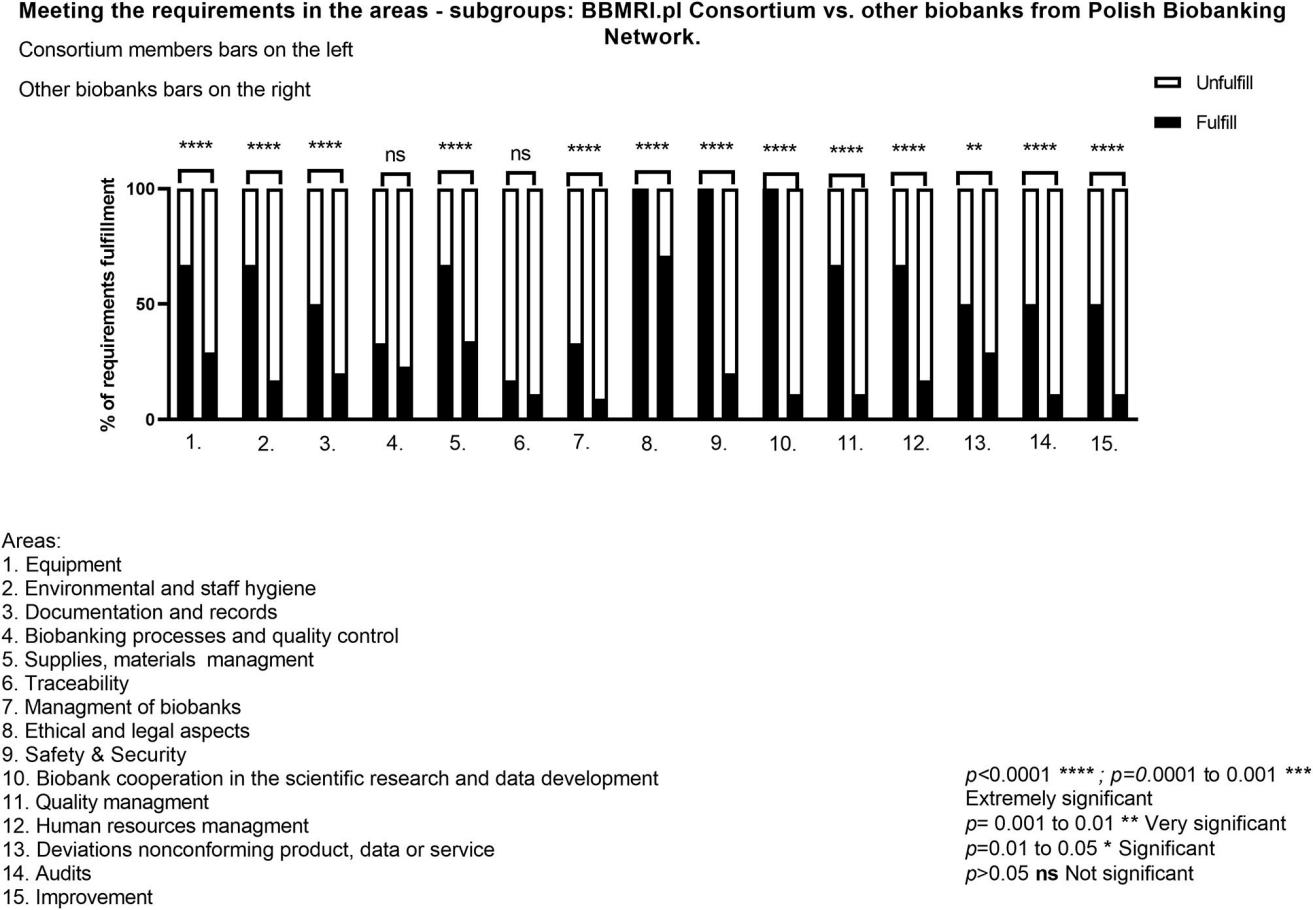
Meeting the requirements in the areas—subgroups: BBMRI.pl Consortium vs. other biobanks from Polish Biobanking Network.

Furthermore, the relationship between the level of QMS implementation and the biobank status in PBN (Member/Observer vs. Consortium member) was examined (Figure 2). The results presented clearly indicate that differences in the fulfillment of the requirements strictly depend on the significance level. It is worth emphasizing that ELSI aspects are the strongest area where the rules are strictly complied with. The areas where the relatively largest discrepancies were noticed between the analyzed subgroups were *Safety&Security* and *Scientific cooperation*.

However, in the areas of *Traceability* and *Biobanking processes and quality control*, no evidence of dependence between the type of biobank membership and the fulfillment of the requirements was observed. Here, being a Member/Observer or a consortium member did not affect the fulfillment of the requirements.

**Figure 3:**
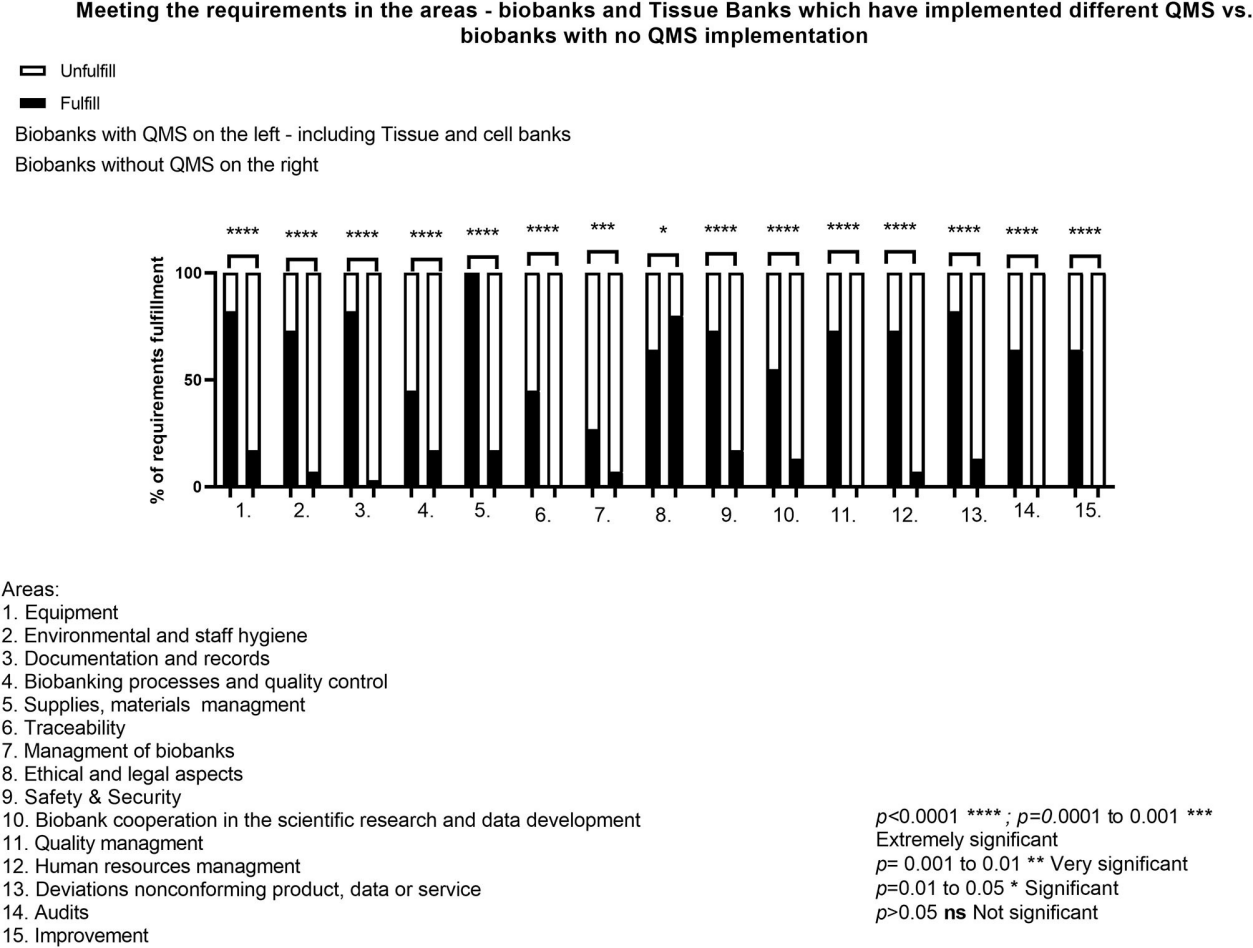
Meeting the requirements in the areas—biobanks and Tissue Banks which have implemented different QMS vs. biobanks with no QMS implementation.

Further analysis focused on the relationship between the biobanks which are located together with Tissue Banks or biobanks with different QMS systems (ISO 9001, ISO 17025) implemented vs. biobanks which did not implement any quality system so far (Figure 3). In the obtained results regarding *p*-value, the statement that extremely significance was estimated in all areas where the requirements comply with ISO 9001:2015 can be found. The strong importance of QMS implementation in all biobanks including Tissue Banks thus can be underlined. Only within the *ELSI*, no relevance was calculated. This might be due to the lack of any specific restrictions in the QMS. Moreover, the level of the fulfillment of the requirement is not determined by the implementation of different QMS, but it strongly depends on the method of obtaining biological material and related data. All statistically important results of the analysis indicate that the presence of the QMS system, compared to the absence of any system, has a great importance. Biobanks with Tissue Banks or any other quality management system, fulfill the requirements in high percentage level, which is as follows: *Equipment-*82%, *Environmental and staff Hygiene* 73%, *Documentation and records, Management of biobanks, Supplies and materials management, ELSI, Traceability, Safety&Security, Scientific Cooperation, Quality Management, Biobanking processes and quality control, Human Resources Management, Audits, Deviations, Incompatible product/data or service, Improvement-*respectively, 82% with the QMS system. It was also postulated that Biobanks with already implemented QMS systems are better prepared to meet the QSPB requirements. A set of SOPs already introduced in the parent organization helps Biobank to fulfill the QSPB documentation and create procedures which could be used as an integrated system. Also Biobank personnel is more aware when their work is already regulated by another quality system. Biobanks with an implemented quality system could also prepare biobank procedures based on the procedures existing in the other quality management system, which also facilitates compliance with the requirements.

The results show that there is a strong correlation between the implementation of the quality system, including the Tissue Banks requirement regarding GMP, and the lack of system implementation. Implementation of a quality system in the biobank's parent unit leads to better preparation of the entity to meet the requirements of the quality system in the specific areas of biobanking. During the audits, it was shown that biobanks which also functioned as Tissue and Cell Banks had a well-educated awareness of the need to develop and implement procedures describing the course of main and auxiliary processes. As a result, biobanks better understood the assumptions of the quality management system dedicated to biobanks and could more easily meet those assumptions. What is important in this context is the fact that a strong, well-developed quality system in Tissue and Cell Banks could be directly integrated with the arising quality system for biobanks. As a result, many procedures and forms could function simultaneously within two units. The most important aspect increasing the importance of the implementation of a properly prepared quality system in biobanks is the aspect of readiness for national and international cooperation within scientific projects, commercial use in the pharmaceutical industry and the development of personalized medicine, thanks to the awareness of the need for the implementation and maintaining of QMS system. Also, the implementation of the ISO 9001 or ISO 17025 system in the parent unit meant that the biobank staff was more aware of the processes taking place within the unit and the need to develop and use SOPs for well-maintained management of the biobank. It was also shown that the *p*-value factor is not significant for biobanks with QMS systems other than GMP as compared to biobanks with no QMS system implemented. It could be concluded that the reason for this is the fact that Tissue and Cell Banks, like Biobanks, collect human biological material and the way they function is similar despite differences in the final use of the target product.

**Figure 4:**
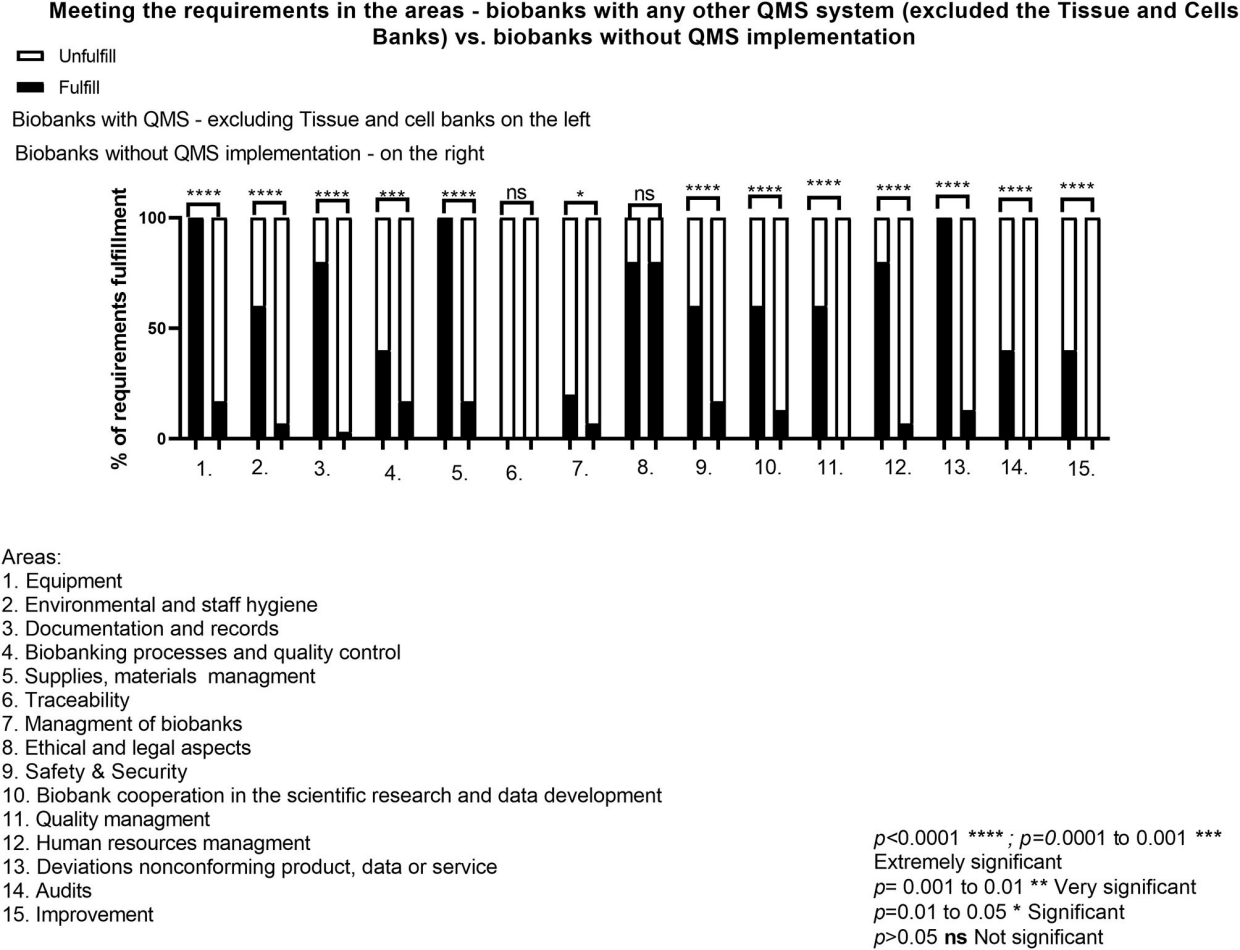
Meeting the requirements in the areas—biobanks with any other QMS system (excluded the Tissue and Cells Banks) vs. biobanks without QMS implementation.

Excluding Tissue Banks from the statement and comparing biobanks with any other QMS system vs. biobanks without QMS, there was no significant *p*-value (>0.05) in the field of *Traceability* and *ELSI* (Figure 4). In other areas, the *p*-value obtained was highly significant. This means that the implementation of the quality system in the organization is crucial for maintaining proper supervision over the areas as well as all system processes and ensuring the adequate quality level to meet the expectations of the customers.

**Figure 5:**
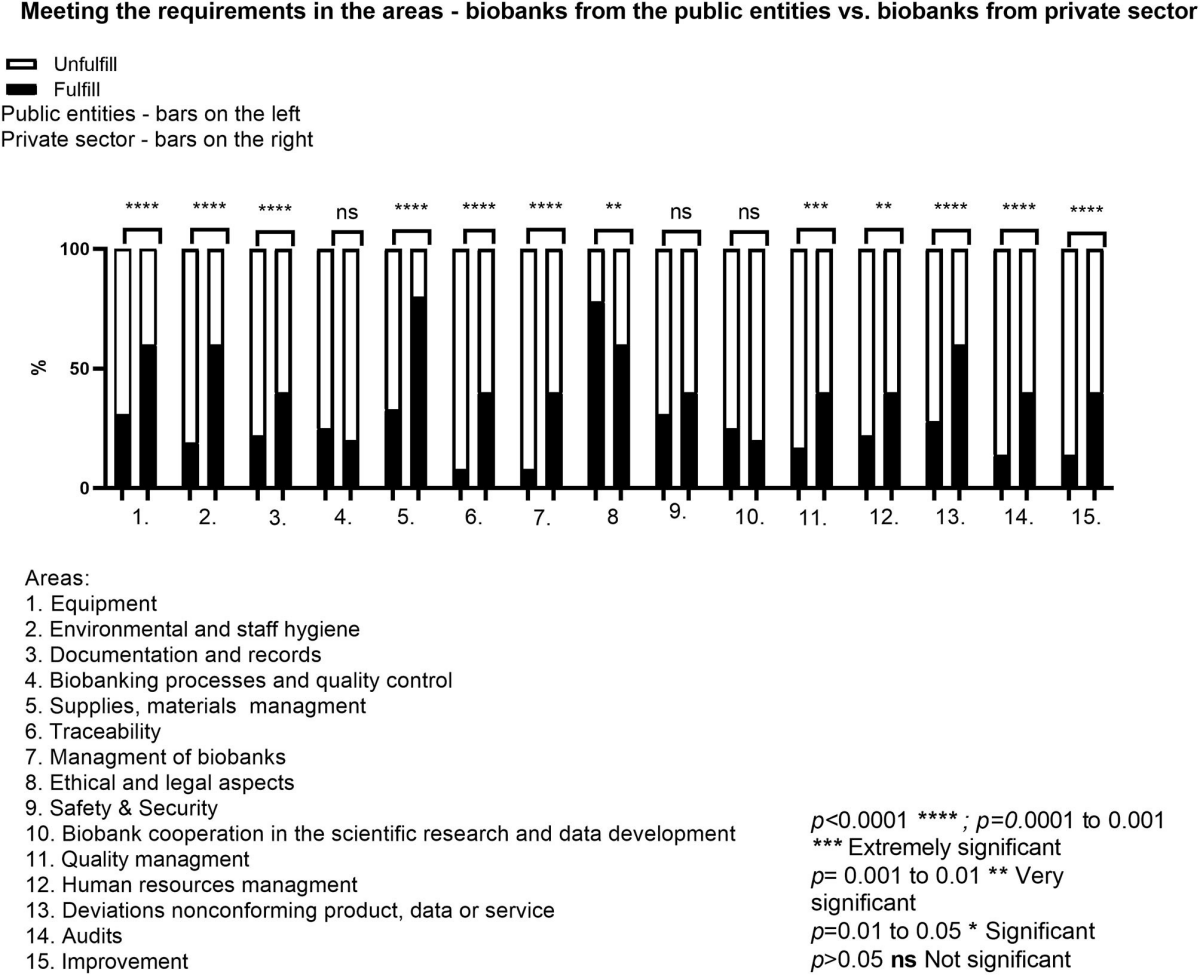
Meeting the requirements in the areas—biobanks from the public entities vs. biobanks from private sector.

The next analysis concerned the comparison of biobanks from the public and private sectors. It was shown that in three cases, there was no significant *p*-value (>0.05)—in the area of *Biobanking processes and quality control, Safety&Security, Scientific Cooperation*. The remaining comparison for the other twelve audited areas showed a high and extremely important statistical significance (*p*-value at rate 0.001–0.01 and <0.0001) (Figure 5). Almost 90% of the biobanks had developed rules for the collection and processing of BM. The information concerning the method of collection, securing and delivery was presented, but not always as documented information. The biobanks also had implemented the procedure for the supervision of non-conformities, which requires that all deviations from the adopted standards, e.g., technological processes, should be registered, analyzed and assessed in terms of potential risks.

For the ELSI area, public biobanks met the requirements to a higher extent than the private ones. The differences concerning the level of compliance with the requirements and implementation of the QMS system in public and private biobanks mainly depend on financial resources and the management of the units. Private entities usually have more resources to hire an adequate number of staff, as well as to prepare and implement certain procedures. Therefore, in private biobanks, a correlation was observed between the following areas: *Deviations/non-conforming product/data/service, Audits, Improvement*. When identified during an internal audit, a non-conformity is removed and a new, better solution is implemented. Also, it is important to “stay on the market” and ensure sustainability. The decisions are more efficient and much faster, especially when the implementation leads to potential scientific cooperation between the biobank and an external unit. No significant *p*-value in meeting the requirements related to *Biobanking processes and quality control, Safety&Security* and *Scientific cooperation* results mainly from the fact that in both public and private units these areas are usually at a similar level of development and their improvement does not depend on the status of the unit.

## Discussion

Comparison with the first summary of 2019 (14) showed a strong trend in ELSI aspects. There was a lower number of identified deficiencies. The verification of Bioethical Committee (BC) approval, donor Informed consent (IC) forms and donor information forms were performed. It was found that over 90% biobank units obtained BC positive opinions for the specific research projects and also IC was preceded before each study performance. Samples data are protected in a dedicated IT system with authorized persons access (password/login). The deficiencies that have been identified: (1) incorrect forms of IC regarding respect for the rights and freedom of a donor (2) lack of records regarding the processing of personal data in accordance with the GDPR, (3) lack of donor's IC, (4) lack of procedures for obtaining IC, (5) lack of procedure in regarding withdrawal of IC and the lack of proceeding in the event of the liquidation of the biobank.

The consciousness of relevance regarding the cooperation between the Data Protection Inspector in the organization, which can influence the improvement of the records which should be implemented on the IC in terms of processing personal data regarding GDPR.

It is worth to point out that on www.bbmri.pl “Code of Conduct on the processing of personal data for the purposes of scientific research by biobanks in Poland,” developed by BBMRI.pl ELSI and IT group is available, where useful information are enclosed for ELSI area improvement.

Moreover, consortium members as model biobanks presented at least the level of 50% of the requirements implementation in most of the audited areas. In contrast, Ferdyn et al. indicated that the most developed areas were *Quality control, Environmental monitoring and hazardous waste handling*. During audits, a positive trend was observed that Biobanks present the highest activity in those three areas and developed other areas where biobanks are most active.

Furthermore, the results obtained in the group comparing the QMS level implementation in the biobanks from BBMRI.pl Consortium and biobanks from PBN indicate some significant differences between biobanks that belong to the BBMRI.pl consortium and other biobanks from the PBN. It is supposed that biobanks belonging to the BBMRI.pl consortium are characterized by a better prepared quality management system because they are located in the parent units that have implemented other quality systems, such as ISO 9001, ISO 17025 or GMP. Moreover, the consortium members are obliged to prepare other biobanks willing to enter the PBN as Members or Observers so that they meet the requirements of the quality management system to enable efficient management of biobanking processes in these units. It is possible to present the hypothesis that biobanks belonging to the BBMRI.pl consortium better meet the QSPB requirements and thus obtain better results during the audit for several important reasons: (1) they have a better developed infrastructure and more resources, (2) they have qualified personnel experienced in the development and implementation of quality systems (3) they have been selected for the consortium due to their high potential to create PBN and thus have an advantage at the start over biobanks outside the consortium.

Audits performed in 2018–2020 brought a lot of relevant information about the status of biobanks. Different levels of QMS implementation have been identified in PBN. The process supervision is highly variable between individual biobanks. Here lies a high potential for improvement and it is a challenge for BBMRI.pl QMS team. Nevertheless, the awareness of the biobank personnel and readiness to improve the QMS and other processes, allows the conclusion that quality assurance is an aspect on which the biobanks want to focus their efforts, using the tools developed as a part of the BBMRI.pl project.

The main goal was to indicate the direction of further harmonized and unified QMS development and improvement, as well as to determine the starting points for QMS development.

All actions prepared within the BBMRI.pl QM Task are carefully designed and planned for a dedicated biobanking unit to unify and implement common solutions in PBN, which are convergent with BBMRI-ERIC general outline. The activities performed were aimed at spreading the idea of biobanking with the assumption that high quality biobank services associated with PBN BBMRI.pl are guaranteed. They are to encourage M/O of PBN to develop biobanking ideas and self-improvement, which will directly result in increased competitiveness, maintaining high position in the industry, reduced unit costs and, above all, improved quality of BM and associated data. It will also enhance the growth of the number and quality of scientific research and R&D carried out in cooperation with foreign scientific centers or enterprises.

The results of the work allow not only for current improvement but also indicate the direction of further activities of BBMRI.pl QM group, including the process of the cross-audit program in Poland. Thanks to the identification of the areas that require special attention, the training offer will be adapted in order to intensify work in these areas. Moreover, it helps to develop the audit's plans in order to increase their effectiveness, through more precise planning of the auditors' working time.

Today, biobanking units are no longer required to give access to BM only, but also to access high-quality material on which repeatable test results can be carried out. Therefore, the role and tasks of the Quality Management processes in biobanking units are subject to continuous development. Biobanks are responsible for building a quality model and creating a competitive, intuitive system that meets the requirements of a wide spectrum of stakeholders.

Our work clearly shows the improvement of the processes and of the quality system itself in biobanks. This is a positive factor for the further development of translational medicine, taking into account the special role of biobanks. Thanks to this, the activities of the biobanks associated in networks as well as the activities of organizations such as BBMRI.pl directly contribute to the improvement of the quality of preclinical research. This is beneficial both for research funding institutions, the healthcare system and, above all, for patients.

## Data Availability Statement

The raw data supporting the conclusions of this article will be made available by the authors, without undue reservation.

## Author Contributions

AM-W, JG-O, and PS: conceptualization. AM-W, PS, ML, MK, KZ, and JG-O: methodology, validation, investigation, and writing—original draft preparation. MK and AM-W: software. AM-W, PS, MK, and KZ: formal analysis. AM-W, PS, ML, KZ, and JG-O: resources and data curation. AM-W, PS, ML, and JG-O: writing—review and editing. AM-W, PS, and MK: visualization. AM-W: supervision, project administration, and funding acquisition. All authors have read and agreed to the published version of the manuscript.

## Funding

The project was financed and supported by the Polish Ministry of Science and Higher Education (DIR/WK/2017/2018/01-1). Organization of Polish Biobanking Network within the Biobanking and Biomolecular Resources Research Infrastructure BBMRI-ERIC.

## Conflict of Interest

The authors declare that the research was conducted in the absence of any commercial or financial relationships that could be construed as a potential conflict of interest.

## Publisher's Note

All claims expressed in this article are solely those of the authors and do not necessarily represent those of their affiliated organizations, or those of the publisher, the editors and the reviewers. Any product that may be evaluated in this article, or claim that may be made by its manufacturer, is not guaranteed or endorsed by the publisher.

## Abbreviations

QMS: Quality Management System
BM: Biological Material
BBMRI-ERIC: Biobanking and BioMolecuar Resources Research Infrastructure
IC: informed consent
QSPB: Quality Standards for Polish Biobanks
ISBER: International Society for Biological and Environmental Repositories
IARC: International Agency for Research on Cancer
ESBB: European Middle Eastern & African Society for Biopreservation and Biobanking
GMP: Good Manufacturing Practice
PBN: Polish Biobanking Network
ISO TC: International Organization for Standardization Technical Committee
SOPs: Standard Operating Procedures.
